# Catching-up: Children with developmental coordination disorder compared to healthy children before and after sensorimotor therapy

**DOI:** 10.1371/journal.pone.0186126

**Published:** 2017-10-11

**Authors:** Mats Niklasson, Torsten Norlander, Irene Niklasson, Peder Rasmussen

**Affiliations:** 1 Center for Research and Development, Evidens University College, Göteborg, Sweden; 2 Center for Sensorimotor Research, Vestibularis Clinic, Kalmar, Sweden; 3 Department of Clinical Neuroscience, Karolinska Institutet, Solna, Sweden; 4 Department of Neuroscience and Physiology, Insitute of Child and Adolescent Psychiatry, Sahlgrenska University Hospital, Göteborg, Sweden; UMR8194, FRANCE

## Abstract

The aims of the present study were to (a) compare healthy children in terms of sensorimotor maturity to untreated children diagnosed with developmental coordination disorder (DCD) and (b) compare healthy children to diagnosed children following completed treatment with sensorimotor therapy. Participants were 298 children, 196 boys and 102 girls, distributed into a Norm group of healthy children (*n* = 99) and a group of children diagnosed with DCD (*n* = 199) with a total mean age of 8.77 years (*SD* = 2.88). Participants in both groups were assessed on instruments aimed to detect sensorimotor deviations. The children in the DCD group completed, during on average 36 months, sensorimotor therapy which comprised stereotypical fetal- and infant movements, vestibular stimulation, tactile stimulation, auditory stimulation, complementary play exercises, gross motor milestones, and sports-related gross motor skills. At the final visit a full assessment was once more performed. Results showed that the Norm group performed better on all sensorimotor tests as compared to the untreated children from the DCD group, with the exception of an audiometric test where both groups performed at the same level. Girls performed better on tests assessing proprioceptive and balance abilities. Results also showed, after controls for natural maturing effects, that the children from the DCD group after sensorimotor therapy did catch up with the healthy children. The concept of “catching-up” is used within developmental medicine but has not earlier been documented with regard to children and youth in connection with DCD.

## Introduction

From a historical perspective poor motor skills among children and youth is not new (e.g., [1–5]). Although it has been more than 100 years since the first reports were published, motor problems and treatment methods are still overlooked within psychiatric research [6]. One reason may be the complexity of the subject [7], and another reason may be that the dialogue between psychiatry and pediatrics is not sufficiently clear [8]. Comorbidity within developmental medicine and child psychiatry is as it were rather the rule than the exception [9]. Research has to an inceasing degree presented associations with learning difficulties [10], problems of concentration, language impairment, behavior problems, overarching academic difficulties (reading and writing), poor social skills, anxiety, depression [11], mental health difficulties, [12] and peer victimization [13]. Studies have also shown that motor difficulties are not outgrown [14, 15].

Over the years motor problems have been labeled in different ways, but ever since 1994 the construct of Developmental Coordination Disorder (DCD) [16–18] has been preferred. It is estimated that the prevalence globally is between 5% och 20%. More boys than girls are affected and those affected may be found both across socio-economic conditions and across cultures [19]. DCD is a neurodevelopmental disorder [20], characterized by a delayed and immature motor ability, which noticeably affects everyday activities. It does not, however, include obvious intellectual or medical causes. Some children with this diagnosis also exhibit a different kind of anomalous motor ability believed to constitute neurodevelopmental immaturities or neurological soft signs (NSS) rather than neurological abnormalities [17]. NSS is a heterogenous mix of movement patterns, some of which are remaining primary reflexes [15] and possibly also defective sensory integration [21]. The European Academy of Childhood Disability (EACD) [19] as well as Wiener-Vacher and colleagues [22] recommended that sensory status (i.e. vestibular function) should be included in the examination conducted when the diagnosis of DCD is determined. Further, as mentioned by Cairney [20], the term ‘developmental’ connotes a nervous system in constant change, which ought to imply possibilities for progress. When it comes to treatment, both the EACD [19] and a recent systematic review and meta-analysis [23] recommended motor- training–based and task-oriented interventions.

At the Vestibularis Clinic in Sweden sensorimotor therapy (SMT) according to the method Retraining for Balance (RB) has been practiced more than 25 years and several reports have shown successful treatment results [15, 24, 25]. The method includes aberrant primary reflex assessment and integration, vestibular assessment and stimulation as well as auditory perceptual assessment and stimulation [24] in order to treat sensorimotor disorders (SMD) in children, adolecents and adults. The concept SMD was tentatively introduced [15] as a complement to the label DCD in order to stress the importance of reentering vestibular function into the diagnostic criteria [26]. Recent research supports this notion through its recognition of vestibular influence upon various aspects of human behavior [27].

The auditory perceptual test examines whether the children have a right- or left-ear dominance or whether dominance is absent. A right-ear advantage (REA) [28] affects school-work e.g. dealing with instructions [29–31], and reduces the sensitivity of the children to non-language sounds (i.e social sounds and noice) [32, 33]. Children are sensitive to noise not the least given that their ability to ignore irrelevant sounds is not yet developed [30, 34] and, not unexpectedly, there is a positive association between high levels of noise, headaches, and fatigue [35]. Motor skills problems are serious threats to both physiologial and psychological health [36, 37] but so far, the role of aberrant primary reflexes, postural reactions, and delayed gross motor milestones beyond infancy has been overlooked in developmental research and the same is true of vestibular function [38]. Empirical data indicate [15, 24, 25] that it is most probable that these motor patterns together with vestibular function are of importance when a child’s nervous system adopts one pathway of development versus another. That observation suggests that children with DCD who also received SMT, despite successful results, will have difficulties attaining the same sensorimotor level as healthy children.

For several years, a few comparison studies based on a larger number of healthy children above 5 years of age have been reported, both with regard to motor performance [39–42] and neuropsychological maturity [43]. In terms of comparisons between healthy and diagnosed children, Konicarova and co-workers in three small samples [44–46] examined the presence of four primary reflexes in both healthy children aged 8- to 11- years and untreated children in the same age range who were diagnosed with ADHD. Furthermore, in a larger intervention study [47] the association between an aberrant asymmetrical tonic neck reflex (ATNR) and reading difficulties was examined. However, to our knowledge no studies exist that compared healthy children to untreated children diagnosed with DCD in terms of a battery of primary reflexes, gross motor skills, and vestibular function. Also, as far as we know, studies are lacking comparing healthy children to children diagnosed with DCD who have undergone treatment.

In order to widen the window of knowledge, a study to collect normative data on preschool- and school children was conducted. The aims of the present study were to (a) compare healthy children of different ages in terms of sensorimotor maturity to untreated children diagnosed with developmental coordination disorder and (b) compare healthy children to diagnosed children following completed treatment with sensorimotor therapy. The study had two hypotheses: (1) The healthy children will perform significantly better on all sensorimotor tests compared to untreated children with developmental coordination disorder, and (2) the improvements expected to be attained by the diagnosed children following sensorimotor therapy will not suffice to catch up with those of the healthy children in terms of sensorimotor performance.

## Method

### Participants

There were 298 children in the study distributed into a Norm group recruited from three schools in a middle-sized city in southeastern Sweden (boys = 49, girls = 50) and a DCD group consisting of children diagnosed with developmental coordination disorder including vestibular disorders (boys = 147, girls = 52). There were four age groups (5 years, 8 years, 10 years, 13 years) with a mean age of 8.77 years. (The Norm group: *M* = 8.66, *SD* = 3.23; DCD group: *M* = 8.82, *SD* = 2.70). Table 1 shows the distribution with regard to age categories and gender.

**Table 1:** Gender distribution over groups and categories.

	Norm group		DCD group	
Category	Boys	Girls	Boys	Girls
5 years	20	12	29	12
8 years	11	14	55	15
10 years	4	7	27	11
13 years	14	17	36	14
Total	49	50	147	52

### Design

All participants in both groups were assessed on three instruments aimed to detect sensorimotor deviations: (1) Retraining for Balance–Physiological Test, (2) Retraining for Balance–Orientation and Balance Test and, (3) Retraining for Balance–Audiometric Test (see Instruments). The children in the Norm group were assessed at their own schools whereas those in the DCD group were assessed at the Vestibularis Clinic. During each assessment either a parent or another adult close to the child was present. As a consequence of their scores on the tests, all participants in the DCD group were diagnosed as having developmental coordination disorder including vestibular disorders. Typically, the children in the DCD group also showed additional problems as assessed, prior to therapy, by their teachers and parents. Most commonly reported difficulties were ‘concentration problems’, ‘mood swings’, ‘reading and writing difficulties’, and ‘social immaturity’. The children in the DCD group completed Sensorimotor Therapy (SMT) using the method Retraining for Balance (RB) [24, 25]. RB comprised seven parts; (a) Stereotypical fetal- and infant movements, (b) Vestibular stimulation, (c) Tactile stimulation, (d) Auditory stimulation, (e) Complementary play exercises, (f) Gross motor milestones, (g) Sports-related gross motor skills. In all, the manual described 48 different exercises, which were used in an adapted sequential order depending on the child’s needs. However, all individual training programs started with fetal movements. During therapy, which lasted on average 36 months, the children practiced about 15 minutes per day at home, together with, or monitored by, their parents. Every 8th week they came back to the Vestibularis Clinic for a review and to learn new exercises. At the final visit, a full assessment was performed which was compared and evaluated in relation to the first assessment.

### Instruments

#### Retraining for Balance-Physiological Test (RB-P)

The Physiological Test [24, 48] was compiled on the basis of research and documentation of the motor development of normal and developmentally delayed children. The battery consisted of 41 different tests and the performances of participants’ were rated on each test on a 5-point scale from 0 to 4 (“No deviation”–“Inability to complete, or execute, a specific item”). The tests were assembled into six groups generating subscales on (1) Primary reflexes-vestibular stimulation, (2) Primary reflexes-tactile stimulation, (3) Postural reactions, (4) Gross motor milestones, (5) Eye movements, and (6) Sports-related gross motor skills. An index was computed for each group by multiplying the mean by 10, yielding a scale with anchors of 0: “No deviation” and 40: “Significant deviation”. The six subscales were then summed to a total value for the Physiological Test. The instrument has acceptable psychometric properties [24] in regard to internal consistency and significant correlations with other sensorimotor instruments.

#### Retraining for Balance-Orientation and Balance Test (RB-OB)

This test [24, 49] consisted of balance and vestibular assessments, which responded to either “No deviation” or “Inability to complete or execute a specific item”. The assessments were assembled in three categories, (a) Standing balance, (b) Vestibular test, and (c) Body-space perception and a mean was computed for the results in each category, and then the categories were summed. A previous study (24) indicated acceptable psychometric properties for Orientation and Balance Test.

#### Retraining for Balance-Audiometric Test (RB-A)

The current test was an auditory-perceptual test, based on a technique developed by Johansen [50], which used the clinical diagnostic audiometer DA 74 (Danaplex, Copenhagen, Denmark). Niklasson et al. [24] focused on the auditory preference in binaural pure tone audiometry and therefore constructed a scale (RB-A) in order to measure whether the particular participant had a right or left ear preference or whether preference was lacking. The scale spanned 0–200, on which values below 100 indicated left-ear dominance, and values above 100 indicated right-ear dominance. Right ear dominance was supposed to facilitate a more rapid processing of speech sounds [29]. The test’s rationale for importance of right ear-dominance was validated by Tallal, Miller, and Holly Fitch [51] and by Okamoto, Stracke, Ross, Kakigi, and Pantev [33].

#### Teacher Rating Scale (TRS)

Conners′ test for teachers [52, 53] was used in a short Swedish version [54, 55]. This version consisted of 27 statements and yielded a summary measure and four subscales, (a) Behavioral problems, (b) Impulsivity or Hyperactivity, (c) Concentration problems, and (d) Inattention. Each statement was checked by the teachers, at the start and at the completion of therapy, on a four-point scale, 0 = “Not at all true”, 1 = “Somewhat true”, 2 = “Quite true”, 3 = “Definitely true”, where “0” indicates no problem and “3” major problems. A TRS total score was computed through averaging values from the sub-scales.

#### Parent Symptom Questionnaire (PSQ)

A shortened, Swedish version [56], (C. Gillberg, personal communication, April 2007) of Conners′ test for parents with children having attentional problems was used [53, 57, 58]. This version consisted of 10 statements with a special focus on attentional variables, which might indicate that the child had ADHD [56]. It yielded a composite measure and three subscales, (a) Behavioral problems, (b) Impulsivity or Hyperactivity, and (c) Inattention. Each statement was checked by the parents at the start and at completion of therapy on four-point scales similar to the Teacher Rating Scale.

#### Reasons for Training (RFT)

A questionnaire [24, 59], which assessed the improvement of the children regarding additional problems, was given to the parents. Before therapy they indicated at most five of their child’s additional problems in order of severity. After therapy the parents rated how much they estimated that each problem had changed according to a 4-point scale with anchors of 0: No positive change, 1: Little positive change, 2: Quite some positive change, 3: Great positive change. The 4-point scale has been validated [24] through comparisons with the Parent Symptom Questionnaire [58].

### Procedure

#### The Norm group

At the start-up of the current study, the head masters of four different school districts in a middle-sized town in the southeast part of Sweden were asked if they were willing to allow interested teachers and children to sign up voluntarily for participation in the study. They were all positively inclined and suggested that interested teachers could sign up voluntarily. In the younger group two teachers with a total of 43 children signed up, in the middle group five teachers with 100 pupils distributed between two age groups signed up, 8-year-olds and 10-year-olds, and in the older group three teachers with a total of 70 pupils signed up. The teachers in all the groups asked the parents to attend separate parent meetings, where one of the current authors gave an approximately 45-minute oral presentation of the lay out of the study, and a written summary was also provided. The parents who were present also had the opportunity to ask questions. At one meeting 20 parents with children in one class which belonged to the middle group declined their children’s participation in the study. The day after each parent meeting all relevant schools received a visit, and the study was presented to the children in each class. In total 193 children took part in the presentations which took approximately 20 minutes each. The children, just as the parents before them, were informed of the purpose of the study, what the testing would be like, and the fact that a third person would be present. That person would be an adult whom the children knew and one the school had selected. Furthermore, everyone was informed that there would be no rewards given for the participation. Following the information an informed consent form was handed out. The idea was that the children quietly and calmly could discuss their possible participation with their parents. If they wished to participate, both children and and parents would sign the form and return it to the school. No exclusion criteria were given either to the children or the parents. A total of 110 children declined participation while 103 children signed up and were tested, but four of them were excluded because they had either trained previously or were currently enrolled at the Vestibularis Clinic. No participant was questioned about nor indicated that they had a potential neuropsychiatric diagnosis. Prior to deciding on the time and place of the testing, a contract was drawn up with the head master of each school who also offered a room where the tests were carried out. The children left their activities or periods for approximately 1 hour in order to participate. All testing sessions were conducted in the same way and aimed to be similar to the testing routines used at the Vestibularis Clinic. Data were collected between 11.14.14 and 09.04.15 and all tests were conducted by two experienced and trained sensorimotor therapists.

#### The DCD group

SMT has been practiced at the Vestibularis Clinic for more than 25 years for children and youngsters with attention disorders and motor problems, and more recently also for adults. Typically, parents had heard about the therapy from other parents, preschool or school administrators or from the school health care provision. The very first visit to Vestibularis was always preceded by oral and written information. Then, at the first visit and before assessment, the children and parents were informed that they should feel free to ask questions and also that they were free to leave at any time during the testing. The assessment session lasted about 60 minutes. Following the testing the participants were informed of the results, and of the decision of potential training. The criteria for admission to SMT have been that participants after sensorimotor testing are diagnosed as having developmental coordination disorder including vestibular disorders [15, 24]. When the therapy was considered completed about three years later, the participant was free from training for about three months. Thereafter, the final sensorimotor assessments were performed and the results were compared to the initial values. Following approval by the parents or adult participants, the report of the client was allowed to be left with Vestibularis to be used as reference for future studies and publications. Since 1999 the Vestibularis Clinic uses the quality management system SS-EN ISO 9001:2008 [60] and within the standard of the system, one controls how data are collected and how files and data are to be handled and stored. Data from all participants in the DCD group in the current study are to be found within the quality system. In order to create a reference group vis-á-vis the healthy children in the current study, previously collected data from children in the same age groups as in the Norm group were used, i.e., the participants in the DCD group were all recruited from children who had undergone Sensorimotor Therapy as a part of the routine clinical care. With the composition of the Norm group as a starting point, there were 199 children who were treated with SMT, in the same age ranges that is the “5-year-olds” group (58–70 months), the “8-year-olds” group (94–107 months) the “10-year-olds” group (125–133 months), and the “13-year-olds” group (144–168 months). These age ranges coincided with a traditional children-and youth division [61].

### Ethical considerations

Prior to the time when the study was conducted, it had been approved by the Regional Ethical Board of Uppsala. The study followed the ethical standards of the World Medical Association’s Declaration of Helsinki concerning Ethical Principles of Medical Research Involving Human Subjects. Written informed consent was obtained from the parents on behalf of both the healthy children and the diagnosed children enrolled in the study.

### Statistical approach

In Results *Section A*, healthy children (Norm group) were compared to untreated children diagnosed with sensorimotor dysfunction (DCD group) with regard to the results of the sensorimotor tests (i. e., RB-P, RB-O and RB-A). Since the number of both boys and girls was less than ten in one of the Norm group age catgory cells, the results from the three-way multivariate analyses were checked with non-parametric statistics (Mann-Whitney and Kruskal-Wallis, 5% level). In *Section B* the treatment results for the DCD group were analyzed in terms of teacher and parent assessments as well as results on the sensorimotor tests. Finally in Results *Section C* we investigated whether or not there were differences between the healthy children from the Norm group and the diagnosed children from the DCD group *after* treatment in regard to total score of the sensorimotor tests. Subsequently tests were performed in order to control for natural maturing effects during treatment time (about three years) through a procedure where the after treatment records of the 5-year-olds group (now a group of 8-year-olds) from the DCD group were compared with the 8-year-olds group from the Norm group. Likewise, a comparison was made between the 10-year-olds group from the DCD group after treatment (now a group of 13-year-olds) and the 13-year-olds group from the Norm group. In order to facilitate an over-view of the results, Cohen’s *d* was calculated for comparisons between the healthy children and the children diagnosed as having DCD. In the after treatment comparisons the *d-*statistics were adapted according to the controls for maturing effects during treatment time. The statistical basis for the current study can be found in the Supporting information section as S1 File, ‘Catching-up. Niklasson et al.sav’.

## Results

### Section A: Comparisons between children in the Norm group and untreated children from the DCD group with sensorimotor disorder

#### Retraining for Balance—Physiological Test

A three-way Pillais' MANOVA (2 x 2 x 4 factorial design) was applied with Group (norm, DCD), Gender (boys, girls) and Age Category (5, 8, 10, 13) as independent variables. The dependent variables were the subscales of the Physiological Test (i. e., Primary reflexes-vestibular stimulation, Primary reflexes-tactile stimulation, Postural reactions, Gross motor milestones, Eye movements, Sports related motor skills) and the total score. The analyses yielded significant effects for Group (*p* < 0.001, *Eta*^*2*^ = 0.50, *power* > 0.99), Gender (*p* = 0.002, *Eta*^*2*^ = 0.08, *power* = 0.96), and Age Category (*p* < 0.001, *Eta*^*2*^ = 0.09, *power* > 0.99). There were no interaction effects (*ps* > 0.05). The results of the univariate F-tests with regard to Group, Gender and Age Category are shown below. Controls with non-parametric statistics (Mann-Whitney and Kruskal-Wallis, 5% level) yielded no other significant indications.

**Group.** Univariate F-tests yielded significant effects for Primary reflexes-vestibular stimulation [*F* (1, 281) = 182.56, *p* < 0.001], Primary reflexes-tactile stimulation [*F* (1, 281) = 30.68, *p* < 0.001], Postural responses [*F* (1, 281) = 105.66, *p* < 0.001], Gross motor milestones [*F* (1, 281) = 192.01, *p* < 0.001], Eye movements [*F* (1, 281) = 72.97, *p* < 0.001], Sports related gross motor skills [*F* (1, 281) = 39.05, *p* < 0.001], and for the total score [*F* (1, 281) = 221.47, *p* < 0.001]. Descriptive analyses showed that in all cases the children’s physiological performance in the Norm group was substantially better as compared to the children in the DCD group. Means and standard deviations are shown in Tables 2 and 3.

**Table 2:** Means (*M*) and standard deviations (*SD*) for age categories regarding Primary reflexes-vestibular stimulation (A), Primary reflexes-tactile stimulation (B), Postural responses (C), Gross motor milestones (D), Eye movement (E), Sports related gross motor (F), and the total score for the Physiological test (RB-P) in the Norm group.

	Physiological test: Norm group									
	5 yr.		8 yr.		10 yr.		13 yr.		All	
	*M*	*SD*	*M*	*SD*	*M*	*SD*	*M*	*SD*	*M*	*SD*
A	6.04	4.27	4.09	3.95	3.50	2.80	2.23	2.69	4.08	3.87
B	1.30	1.93	0.40	1.11	0.30	1.01	0	0	0.56	1.37
C	3.18	4.29	2.40	2.94	2.40	2.48	1.44	1.90	2.35	3.19
D	7.81	6.71	4.20	5.98	2.27	3.25	2.26	2.36	4.55	5.62
E	15.81	10.74	10.00	10.74	4.09	3.68	2.69	3.98	8.93	10.08
F	13.96	9.88	5.50	7.71	0.45	1.01	2.20	3.34	6.64	8.79
RB-P	48.11	23.98	26.59	24.96	13.01	9.85	10.82	10.23	27.10	25.03

*Note*: Higher scores on the RB-P indicate worse performance.

**Table 3:** Means (*M*) and standard deviations (*SD*) for age categories regarding Primary reflexes-vestibular stimulation (A), Primary reflexes-tactile stimulation (B), Postural responses (C), Gross motor milestones (D), Eye movement (E), Sports related gross motor (F), and the total score for the Physiological test (RB-P) in the DCD group.

	Physiological test: DCD group (before treatment)									
	5 yr.		8 yr.		10 yr.		13 yr.		All	
	*M*	*SD*	*M*	*SD*	*M*	*SD*	*M*	*SD*	*M*	*SD*
A	18.24	7.59	16.65	6.97	13.04	5.47	11.07	5.55	14.89	7.04
B	4.62	5.94	3.84	5.08	4.34	4.99	3.13	3.89	3.92	4.98
C	14.92	7.94	13.70	8.49	8.73	7.36	6.68	5.06	11.22	8.10
D	18.54	8.88	17.58	7.23	16.36	7.69	13.95	7.34	16.63	7.84
E	26.24	11.90	23.93	10.16	17.17	8.40	11.52	7.55	20.00	11.19
F	13.04	4.18	18.95	11.10	13.60	9.68	8.87	7.18	14.18	9.60
RB-P	94.78	27.02	94.64	33.57	73.25	27.09	55.22	24.71	80.61	33.37

*Note*: Higher scores on the RB-P indicate worse performance.

**Gender.** Univariate F-tests showed only one significant effect, i. e., Eye movements [*F* (1, 281) = 6.81, *p* = 0.010] which obtained a pattern where the girls performed better (*M* = 11.85, *SD* = 11.33) than the boys (*M* = 18.64, *SD* = 11.72).

**Age category.** Univariate F-tests yielded significant effects for Primary reflexes-vestibular stimulation [*F* (3, 281) = 7.96, *p* < 0.001], Postural responses [*F* (3, 281) = 6.13, *p* < 0.001], Gross motor milestones [*F* (3, 281) = 4.88, *p* = 0.003], Eye movements [*F* (3, 281) = 28.36, *p* < 0.001], Sports related gross motor skills [*F* (3, 281) = 12.23, *p* < 0.001] and for the total score [*F* (3, 281) = 23.70, *p* < 0.001]. Post hoc testing (one-way ANOVA and Tukey HSD, 5% level) indicated a main pattern where the children tended to enhance their performance for each age level on sub-tests and total scores. Means and standard deviations are shown in Tables 2 and 3.

#### Retraining for Balance—Orientation and Balance Test

A three-way ANOVA (2 x 2 x 4 factorial design) was conducted with Group (norm, DCD), Gender (boys, girls) and Age Category (5, 8, 10, 13) as independent variables. The dependent variable was the Orientation and Balance test. The analyses yielded a significant effect for Group [*F* (1, 282) = 405.54, *p* < 0.001, *Eta*^*2*^ = 0.59, *power* > 0.99] where descriptive analysis showed that the Norm group performed better compared to the DCD group. There was a significant effect for Gender [*F* (1, 282) = 10.08, *p* = 0.002, *Eta*^*2*^ = 0.04, *power* = 0.89] and subsequent analyses showed that girls (*M* = 1.25, *SD* = 0.90) performed somewhat better than boys (*M* = 1.90, *SD* = 0.86). There was also a significant effect for Age Category [*F* (3, 282) = 30.55, *p* < 0.001, *Eta*^*2*^ = 0.25, *power* > 0.99] indicating that the 13-year-olds category performed better on the Orientation and Balance test as compared to the other age groups. Finally, there was a significant effect for Group x Age Category interaction [*F* (3, 282) = 9.04, *p* < 0.001, *Eta*^*2*^ = 0.09, *power* > 0.99] where a post hoc test (Independent Samples *t*-test, 5% level) indicated regarding the Norm group no difference between the 10-year-olds and the 13-year-olds, while the 5-year-olds and 8-year-olds showed significant differences both between themselves and the other two age categories. The analyses concerning the DCD group revealed that the 13-year-olds performed significantly better than the other three age categories, but there were no significant differences between themselves. Controls with non-parametric statistics (Mann-Whitney and Kruskal-Wallis, 5% level) yielded no other significant indications. Means and standard deviations for Group and Age Category are shown in Tables 4 and 5.

**Table 4:** Means (*M*) and standard deviations (*SD*) for age categories regarding Orientation and Balance Test (RB-OB) and the Audiometric Test (RB-A) in the Norm group.

	RB-OB and RB-A: Norm group									
	5 yr.		8 yr.		10 yr.		13 yr.		All	
	*M*	*SD*	*M*	*SD*	*M*	*SD*	*M*	*SD*	*M*	*SD*
RB-OB	1.31	0.61	0.86	0.68	0.14	0.23	0.16	0.35	0.71	0.73
RB-A	104.41	30.01	100.76	40.30	110.00	25.22	114.48	35.14	107.26	34.00

*Note*: Higher scores on the RB-O indicate worse performance. Higher scores on the RB-A indicate better performance (values above 100 indicate right-ear dominance).

**Table 5:** Means (*M*) and standard deviations (*SD*) for Age Categories regarding Orientation and Balance Test (RB-OB) and the Audiometric Test (RB-A) in the DCD group before (pre) and after (post) treatment.

	RB-OB and RB-A: DCD group (before and after treatment)									
	5 yr.		8 yr.		10 yr.		13 yr.		All	
	*M*	*SD*	*M*	*SD*	*M*	*SD*	*M*	*SD*	*M*	*SD*
RB-OB Pre	2.24	0.27	2.34	0.60	2.22	0.52	1.80	0.60	2.16	0.57
RB-OB Post	0.77	0.61	0.80	0.72	0.52	0.63	0.34	0.54	0.62	0.66
RB-A Pre	101.20	27.90	102.16	32.93	108.97	27.80	102.59	24.93	103.44	29.02
RB-A Post	126.20	24.26	130.84	28.26	134.48	27.14	117.26	30.62	127.01	28.45

*Note*: Higher scores on the RB-O indicate worse performance. Higher scores on the RB-A indicate better performance (values above 100 indicate right-ear dominance).

#### Retraining for Balance—Audiometric Test

In order to examine right dominant hearing based on an interval scale, a three-way ANOVA (2 x 2 x 4 factorial design) was conducted with Group (norm, DCD), Gender (boys, girls) and Age Category (5, 8, 10, 13) as independent variables. The dependent variable was the Audiometric Test. The analyses yielded no significant effects (*ps* > 0.05). Controls with non-parametric statistics (Mann-Whitney and Kruskal-Wallis, 5% level) yielded no other significant indications. Means and standard deviations for Group and Age Category are shown in Tables 4 and 5.

### Section B: Effects of treatment in regard to the DCD group

#### Teachers’ and parents’ assessments

The 199 children in the DCD group were all treated with SMT for about three years (*M* = 40.38 months, *SD* = 16.42) when they visited the Vestibularis Clinic on average on 16.88 occasions (*SD* = 4.61). Before treatment, the situations of the children were assessed by teachers from their schools with Teacher Rating Scale (TRS) and by their parents with Parent Symptom Questionnaire (PSQ). After finishing treatment with SMT, teachers and parents once more had to complete the instruments. Paired Samples t-test (5% level) showed for TRS a significant difference [*t* (68) = 4.94, *p* < 0.001] before (*M* = 0.88, *SD* = 0.58) and after (*M* = 0.56, *SD* = 0.46) treatment. Likewise analyses showed a significant effect for PSQ [*t* (85) = 10.04, *p* < 0.001] before (*M* = 1.22, *SD* = 0.68) and after (*M* = 0.50, *SD* = 0.43) treatment. Further, after treatment the parents had to complete the instrument Reasons For Training (RFT) where they checked on a four-point scale the extent of positive change for the main reason for participation. Of the parents, 68 (34.2%) indicated”Great positive change”, 90 (45.2%)”Quite some positive change”, 13 (5.5%)”Little positive change”, and 3 parents (1.5%)”No positive change”. Of the parents 25 (12.6%) did not complete the questionnaire. There were no differences with regard to Age group (Kruskal-Wallis, *p* = 715) or Gender (Mann-Whitney, *p* = 0.661) regarding perception of positive changes.

#### Retraining for Balance—Physiological Test

A three-way mixed Pillais’ MANOVA was conducted with the children from the DCD group where Treatment (before, after) was the within-subjects factor and Age Category (5, 8, 10, 13), Gender (boys, girls) were the between-subjects factors. The dependent variables were the subscales of the Physiological Test and the total score. The analyses yielded significant effects for Treatment (*p* < 0.001, *Eta*^*2*^ = 0.85, *power* > 0.99), Age Category (*p* < 0.001, *Eta*^*2*^ = 0.11, *power* > 0.99), Gender (*p* < 0.001, *Eta*^*2*^ = 0.15, *power* > 0.99) and for Treatment x Age Category interaction (*p* < 0.001, *Eta*^*2*^ = 0.11, *power* > 0.99). There were no other significant effects (*ps* > 0.05). The results of the univariate F-tests with regard to Treatment showed that the children significantly improved their sensorimotor abilities on all sub-scales as well as total score for the Physiological Test. Concerning Age Category univariate F-tests showed significantly effects for all dependent variables with the exception for Primary reflexes-tactile stimulation where the over-all pattern indicated (Tukey-HSD, 5% level) no significant differences between 5- and 8-year-olds categories as well as no differences between 10- and 13-year-olds categories but the two categories with older children performed better as compared to the two categories with younger children. The significant effect for Gender concerned gross motor milestones and eye movements where girls performed better as compared to boys. Finally, the interaction effect was explained (Independent Samples *t*-test, 5% level) by a pattern indicating that the children in the 5-year-olds category often had at better results at base line compared to children in the 8-year-olds category. For means and standard deviations, see Table 6.

**Table 6:** Means (*M*) and standard deviations (*SD*) for age categories regarding Primary reflexes-vestibular stimulation (A), Primary reflexes-tactile stimulation (B), Postural responses (C), Gross motor milestones (D), Eye movement (E), Sports related gross motor (F), and the total score for the Physiological test (RB-P) in the DCD group before treatment (pre) and after treatment (post).

	Physiological test: DCD group (before and after treatment)									
	5 yr.		8 yr.		10 yr.		13 yr.		All	
	*M*	*SD*	*M*	*SD*	*M*	*SD*	*M*	*SD*	*M*	*SD*
A1	18.24	7.59	16.65	6.97	13.04	5.47	11.07	5.55	14.89	7.04
A2	2.53	2.67	1.82	1.98	1.08	1.57	0.76	1.39	1.56	2.04
B1	4.62	5.94	3.84	5.08	4.34	4.99	3.13	3.89	3.92	4.98
B2	1.41	3.53	0.42	1.21	0.81	2.50	0.46	1.09	0.71	2.15
C1	14.92	7.94	13.70	8.49	8.73	7.36	6.68	5.06	11.22	8.10
C2	0.88	1.87	1.32	2.55	0.88	1.68	0.89	1.62	1.04	2.04
D1	18.54	8.88	17.58	7.23	16.36	7.69	13.95	7.34	16.63	7.84
D2	2.01	3.92	1.46	2.99	0.88	2.19	0.30	0.96	1.17	2.78
E1	26.24	11.90	23.93	10.16	17.17	8.40	11.52	7.55	20.00	11.19
E2	3.72	4.55	2.10	4.12	1.23	1.93	0.30	0.99	1.81	3.53
F1	13.04	4.18	18.95	11.10	13.60	9.68	8.87	7.18	14.18	9.60
F2	4.81	5.85	3.05	5.94	1.11	3.52	0.79	1.94	2.47	4.98
RB-P Pre	94.78	27.02	94.64	33.57	73.25	27.09	55.22	24.71	80.61	33.37
RB-P Post	15.36	14.77	10.17	12.92	5.99	8.01	3.50	4.25	8.76	11.70

*Note*: Higher scores on the RB-P indicate worse performance.

#### Retraining for Balance—Orientation and Balance Test

A three-way mixed ANOVA was conducted with the children from the DCD group where Treatment (before, after) was the within-subjects factor and Age Category (5, 8, 10, 13), Gender (boys, girls) were the between-subjects factors. The dependent variable was the Orientation and Balance Test. The analyses yielded significant effects for Treatment [*F* (1, 190) = 570.78, *p* < 0.001, *Eta*^*2*^ = 0.75, *power* > 0.99], for Age Category [*F* (3, 190) = 9.87, *p* < 0.001, *Eta*^*2*^ = 0.14, *power* > 0.99] and for Gender [*F* (1, 190) = 15.88, *p* < 0.001, *Eta*^*2*^ = 0.08, *power* = 0.98]. Descriptive analyses showed that the children’s results improved in terms of orientation and balance on the basis of the Orientation and Balance Test during the treatment period. Concerning Age Category a post hoc test (Tukey-HSD, 5% level) showed the same pattern as for the Physiological Test, i. e., no significant differences between 5- and 8-year-olds categories as well as no differences between 10- and 13-year-olds categories but the two categories with older children performed better as compared to the two categories with younger children. The significant gender difference was due to that girls performed better (Before: *M* = 1.94, *SD* = 0.43: After: *M* = 0.41, *SD* = 0.56) as compared to boys (Before: *M* = 2.24, *SD* = 0.59: After: *M* = 0.70, *SD* = 0.68). There were no other significant results (*ps* > 0.05). For further means and standard deviations see Table 5.

#### Retraining for Balance—Audiometric Test

In order to examine the improvements concerning right dominant hearing based on an interval scale, a three-way mixed ANOVA was conducted with the children from the DCD group where Treatment (before, after) was the within-subjects factor and Age Category (5, 8, 10, 13), Gender (boys, girls) were the between-subjects factors. The dependent variable was the Audiometric Test. The analyses yielded a significant effect for treatment [*F* (1, 159) = 62.24, *p* < 0.001, *Eta*^*2*^ = 0.28, *power* > 0.99]. Descriptive analyses showed that the children’s results on the Audiometric Test improved during the treatment period. There were no other significant effects (*ps* > 0.05). For means and standard deviations see Table 5.

### Section C: Healthy children in comparison to children treated with sensorimotor therapy (SMT)

#### Retraining for Balance—Physiological Test

**Fig 1:**
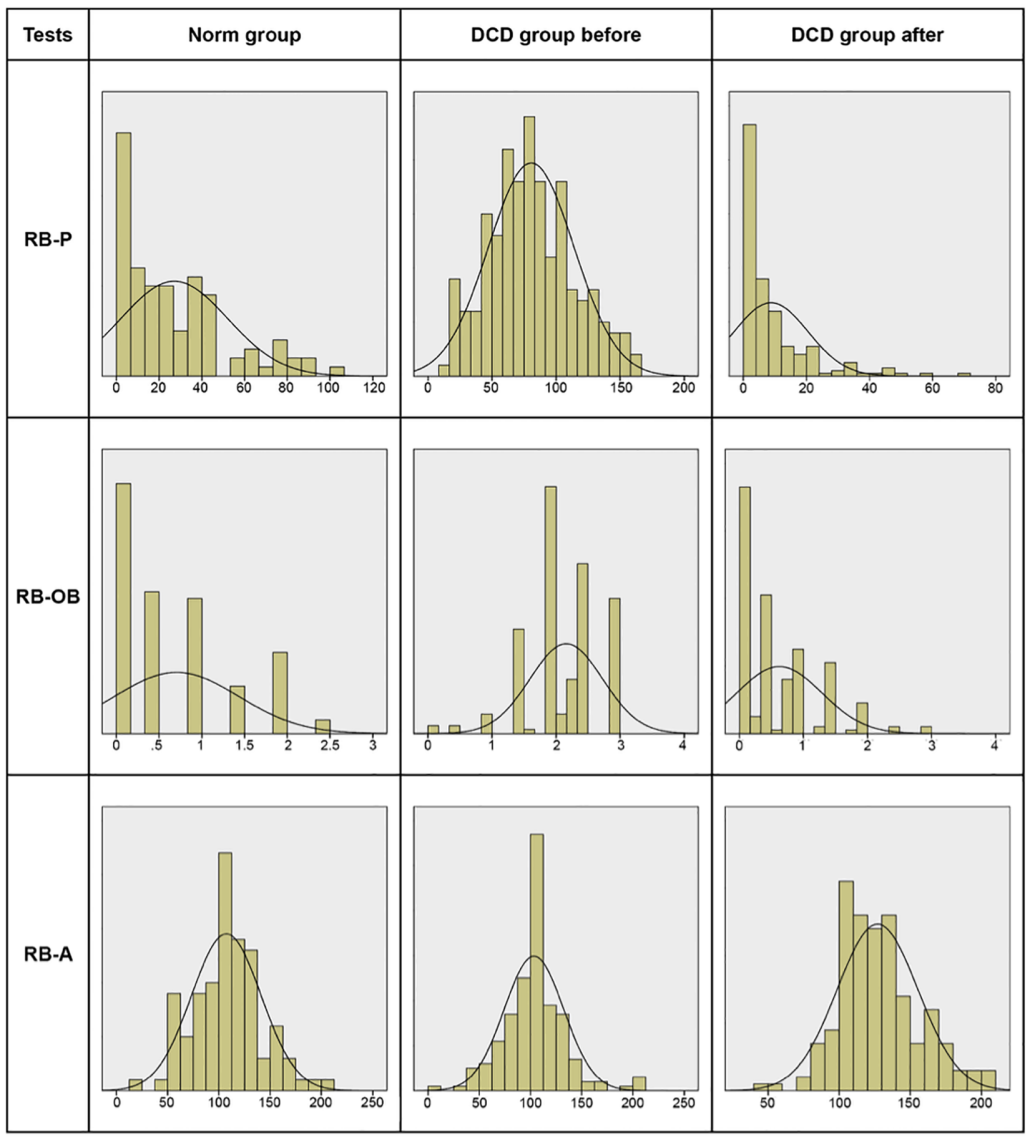
Distribution of raw scores in relation to the curve of normal distribution for the Physiological test (RB-P), the Orientation and Balance test (RB-OB) and the Audiometric test (RB-A) in regard to the Norm group and the DCD group before and after treatment.

The results of the Norm group were compared to the results of the DCD group after treatment through Paired Samples t-tests (5% level). Results showed [*t* (98) = 5.99, *p* < 0.001] that the children in the Norm group did not perform as well (*M* = 27.10, *SD* = 25.03) as the treated children in the DCD group did (*M* = 8.76, *SD* = 11.70). Controls for natural maturing effects during treatment time were performed in regard to the 8-year-olds from the Norm group and treated children of same age from the DCD group, as well as the 13-years-olds from both groups. The first comparison (Independent Samples t-test, 5% level) yielded a significant effect [*t* (64) = 2.30, *p* = 0.025] for Physiological Test where the 8-year-olds in the Norm group did not perform as well as the treated 8-year-olds in the DCD group. The second comparison showed similar results [*t* (128) = 4.74, *p* < 0.001] and conclusions. For means and standard deviations after maturing controls, see Table 7. For Cohen’s *d* in both before and after treatment comparisons see Table 8. Distributions of the tests’raw scores are presented in Fig 1.

**Table 7:** Comparisons between 8 years old and 13 years old children from the Norm group and children of same ages (i. e., the initial 5 and 10 years age categories) from the DCD group after treatment in regard to the Physiological test (RB-P), the Orientation and Balance test (RB-OB) end the Audiometric test (RB-A).

	Norm group				DCD group (after treatment)			
	8 yr.		13 yr.		8 yr.		13 yr.	
	*M*	*SD*	*M*	*SD*	*M*	*SD*	*M*	*SD*
RB-P	26.59*	24.96	10.82*	10.23	15.36*	14.77	5.99*	8.01
RB-OB	0.86	0.68	0.16*	0.35	0.77	0.61	0.52*	0.63
RB-A	100.76*	40.30	114.48	35.14	126.20*	24.26	134.48	27.14

*Note*: Higher scores on the RB-P and RB-O indicate worse performance. Higher scores on the RB-A indicate better performance (values above 100 indicate right-ear dominance).

*Note*: Significant differences between Norm and DCD groups are market (*) in the two conditions.

**Table 8:** Cohen’ *d* for comparisons between healthy children (Norm) and children diagnosed as having DCD in regard to the Physiological test (RB-P), the Orientation and Balance test (RB-OB) and the Audiometric test (RB-A) before and after treatment for the diagnosed children.

	RB-P	RB-OB	RB-A	Mean *d*
Norm vs DCD (before treatment)	+ 1.83	+ 2.23	+ 0.12	+ 1.39
Norm vs DCD (after treatment)	- 0.55	+ 0.37	- 0.72	- 0.30

*Note*: Cohen’s *d* in the after treatment comparison has been adapted according to controls for maturing effects during treatment time.

*Note*: (+) indicate that the children in the Norm group performed better and (–) indicate that the children in the DCD group performed better.

*Note*: The mean *d* for the two motor tests (RB-P and RB-OB) indicated in the before treatment comparison + 2.03 and in the after treatment comparison -0.09.

#### Retraining for Balance—Orientation and Balance Test

In regard to performance on the Orientation and Balance Test analysis (Paired Samples t-test, 5% level) did not show a significant difference between the children in the Norm group and the children in the DCD group after treatment (*p* > 0.05). Controls for maturing effects during treatment time (Independent Samples t-test, 5% level) indicated no significant difference in regard to the 8-year-olds from the Norm group and treated children of same age from the DCD group (*p* > 0.05). Concerning the 13-year-olds subsequent analysis showed [*t* (128) = −2.72, *p* = 0.007] that the children from the Norm group performed better as compared to the treated children in the DCD group. For means and standard deviations after maturing controls, see Table 7. For Cohen’s *d* in both before and after treatment comparisons see Table 8. Distributions of the tests’raw scores are presented in Fig 1.

#### Retraining for Balance—Audiometric Test

The results of the Norm group were also compared (Paired Samples t-test, 5% level) to the results of the DCD group after treatment in regard to the Audiometric Test. Results showed [*t* (86) = −4.99, *p* < 0.001] that the Norm group had significant lower results than the DCD group. Note that on the Audiometric Test higher scores indicate better performance. Controls for maturing effects during treatment time (Independent Samples t-test, 5% level) indicated a significant difference [*t* (58) = 3.05, *p* = 0.030] where the 8-year-olds from the Norm group had significant lower results than the treated 8-year-olds from the DCD group. Concerning the comparison between the 13-years-olds there was no significant difference (*p* > 0.05). For means and standard deviations after maturing controls, see Table 7. For Cohen’s *d* in both before and after treatment comparisons see Table 8. Distributions of the tests’raw scores are presented in Fig 1.

## Discussion

The current study had two hypotheses: (1) The healthy children will perform significantly better on all sensorimotor tests compared to untreated children with developmental coordination disorder, (2) the improvements expected by the diagnosed children following sensorimotor therapy will not suffice to catch up with the sensorimotor performance of the healthy children.

Norm data from healthy (i. e., non-diagnosed or “normal”) preschool- and school- children concerning primary reflexes, postural reactions, gross motor milestones, vestibular function and auditory perception were collected in order for the first time to make possible a comparison with untreated children who were diagnosed as having developmental coordination disorder. Results showed, in accordance with the first hypothesis, that the Norm group performed significantly better on all subscales of the Physiological Test as well as on the total score, compared to the DCD group before treatment and this pattern was also evident for the Orientation and Balance Test but there was no significant effect between groups in regard to the Audiometric Test.

The Norm group performed better both on the Physiological Test and the Orientation and Balance Test, and it also constituted an expected result that both groups improved their results with increasing age, although the pattern was more evident in the Norm group. In terms of the Orientation and Balance Test, the 5- and 8 year-olds of the Norm group exhibited significant differences between each other and the other two age categories, but there was no difference between the 10- and 13-year-olds. Similar results were obtained in a study on neurocognitive tasks [43] where dramatic improvements were seen between 6- and 10-years of age. In both groups the 13-year-olds performed the best, but there were no significant differences between the various age groups of the DCD group. The results of the Audiometric Test were surprising. Given that the Norm group had performed better on both the Physiological Test and the Orientation and Balance Test, one might have expected the same pattern with regard to the Audiometric Test. To our knowledge, there does not exist any previous norm study of expected results of the REA (right-ear advantage) in the various age groups, but both groups in the current study exhibited what may be interpreted as a slight right ear advantage, but there were no significant differences between those two groups. One possible reason for the similar results in the two groups may be that children of today spend their time in noisy environments [62, 63].

Concerning gender aspects there was only one significant difference for the Physiological Test on one sub-scale (Eye movements), where girls performed better, which was in line with a previous study [24]. Girls also performed better on the Orientation and Balance Test a result which is in accordance with previous studies of healthy children up to 12 years of age, a finding which shows that girls performed better on tests assessing proprioceptive, vestibular, and visual abilities [64–66]. It is worth noting that the results indicated that the same phenomenon is true even for the girls of the DCD group, prior to SMT. DCD is considered a “boys′ disorder”, although it could be argued that the gender differences are due to how the children were diagnosed [20]. No consensus exists regarding why boys perform more poorly than girls, but some possible causes suggest that boys are more immature and non-motivated [67] and that they only in the testing situation become more inattentive and irritable [65], or that hyperactivity is the cause [68]. Peterson and associates [64] argued that boys and girls differ in that boys are more dynamic in their movements, whereas girls get more involved in activities of a more static quality. That fact may be reflected on the tests. In the present study the tests within Orientation and Balance Test are of a more static quality, possible reflected in the outcome.

Analyses of age categories indicated a main pattern where the children from the Norm group enhanced their performance on the Physiological Test with each age level, while children from the DCD group did not show such a clear picture. Concerning the Orientation and Balance Test results for both the Norm group and the DCD group indicated that the 13-year-olds group performed better as compared to the other age groups. The analyses yielded no significant effects for the Audiometric Test. The results are in line with earlier studies, which showed that both children with motor problems [15, 69, 70] and children without diagnosed motor problems [66, 71] reached the adult level of motor function in early puberty. The positive developmental trend in the Norm group and the delay in the DCD group strenghthens the results, which previously showed that motor problems are not outgrown (e.g. [14, 15]). Even vestibular perception keeps developing up until early puberty [66] a notion that could explain why 13-year-olds in both groups performed better than the others on the Orientation and Balance Test. In terms of the Audiometric Test, a gradual maturation of auditive function is seen throughout childhood, and adulthood level is usually reached prior to the age of 16 [30, 34] A right-ear advantage (REA) is most likely established in both genders as early as at the age of 4 [72].

Even in terms of the second purpose of the study, for the first time we presented comparisons between healthy children (the Norm group) and diagnosed children following completion of treatment with sensorimotor therapy (the DCD group). A direct statistical comparison showed that the children in the Norm group did not perform as well as the treated children in the DCD group did on the Physiological Test and the Audiometric Test, and that there was no difference between groups concerning the Orientation and Balance Test. However, such a direct comparison is not a reasonable one to make, given the effects of maturity shown in the current study. The design used enabled us to control for natural maturity effects during treatment. Those controls showed that children from the DCD group who were 8 and 13 years old following treatment performed significantly better on the Physiological Test compared to children of the same age from the Norm group. In terms of the Orientation and Balance Test the corresponding controls for maturation exhibited no significant difference between the 8-year-olds from the two groups, but the 13-year-olds of the Norm group performed better compared to the treated children in the DCD group. Finally, the controls for maturation regarding the Audiometric Test showed that the treated 8-year-olds from the DCD group attained better results compared to the 8-year-olds of the Norm group, whereas the comparisons regarding the 13-year-olds did not show a significant difference. Possible confounding factors, despite the controls for maturation, might be the test-learning effects i.e. subjective reinforcement [73] for the DCD group, or the notion that some children from the Norm group had a non-diagnosed developmental coordination disorder.

All in all, however, an evaluation of the comparisons between the healthy children from the Norm group and the treated children from the DCD group indicated that the children from the DCD group regarding sensorimotor maturity did catch up (i.e., narrowed the gap) with the healthy children. This observation is confirmed by statistical analysis using Cohen's *d* which showed as expected a very large effect size (*d* = 2.03) between the Norm group and the DCD group concerning the two used motor tests (RB-P and RB-OB) in the untreated comparison, but in the treated comparison the effect size was negligible (*d* = -0.09). According to Cohen [74] an effect-size of 0.20 is to be considered a small effect, 0.50 a medium effect and 0.80 a large effect. This result was surprising and led to the fact that the second hypothesis of the current study was rejected. The concept of”catching-up” has been used within developmental medicine [19, 75–77] with different connotations but as far as we know it has never been documented through sensorimotor assessments (in terms of a battery of primary reflexes, gross motor skills, and vestibular function) before and after treatment with regard to children and youth in connection with DCD. In the present study Holt’s definition [75] i.e., *“Catching up requires development at a quicker rate than normal for a period”* (p.4) was used. Further, we also agree with Holt that the concept “normal” is a descriptive term, which “*can be applied to any child who shows typical characteristics for his age*” (p.4) that is, with respect to some measured characteristic.

The substantial improvements following treatment shown by the DCD group in the current study are in line with the results of previous studies of sensorimotor treatment [15, 47] and indicated that sensorimotor problems can be treated. Teacher and parent assessments as well as results on the sensorimotor tests showed that the 199 children in the DCD group significantly enhanced their performance through sensorimotor therapy (SMT). However, children with motor problems often exhibit additional difficulties. For this reason, the parents indicated, prior to the start of SMT, the additional problems of their children, and then they mentioned, among other things, concentration problems, mood swings, reading and writing difficulties, and social immaturity, a finding in line with problems reported in earlier research (e.g. [20, 78, 79]). At the completion of the training, the parents rated the improvement with regard to additional problems and the results showed that 79% perceived at least “quite some positive change”. In a forthcoming study we plan to analyze the long-term effects of SMT in the current and in other previous cohorts. In terms of the results of the current study, in accordance with the principle of equifinality [80], similar results for the additional problems might have been obtained even following different efforts such as e.g. combinations of adjusted training, behavior modification, and medication. Given that DCD is a multi-faceted phenomenon and often implies comorbidity with various other disorders [81] there must exist more tools in the tool box. At the same time, it is important to discuss and develop realistic treatment goals. Especially within schizophrenia research the concept of remission has been put forward [82–84] but also within other fields [85]. The concept can be described as the patient developing a good enough function in order to function well in society with the help of various strategies. Stabilization at the level of remission with improved quality of life as a consequence also constitutes a better starting point for an even greater recovery [86]. It is possible that the good results on the sensorimotor tests for the children of the DCD group after training indicate that a majority of the children had attained the level of remission. Only future research can answer this question and the question whether the concept of remission will turn out to be of importance to DCD diagnoses.

The present study had some limitations. Due to teachers’ and pupils’ heavy workload it was difficult to get access to classes and the study would of course have benefitted from a larger Norm group. Another limitation was that the pupils’ participation was voluntary which might have implied that both less able and more developed children refused to take part. Further studies are advised to include whole classes. However, it should also be noted that the Norm group in the present study was clearly in line with Norm groups previously put together in terms of motor performance [39–42], the assessment of some primary reflexes [44–47], and the stronger abilities of girls concerning proprioception and balance [64–66].

The current study showed for the first time through a battery of sensorimotor tests, with assessments before and after treatment, that a group of children with developmental coordination disorder was able to catch up with healthy children through a process-oriented [19, 87–89] and parent centered [11, 19, 23] sensorimotor therapy. The study also showed, through teachers′ and parents′ assessments, that the additional problems of the children were reduced in several ways. The current study points to the necessity of continued research both on treatment methods and treatment goals for DCD. The disorder involves a general physical inactivity and physical inactivity is presently regarded as a “worldwide pandemic” [90]. In order to better understand why many children and adults, both diagnosed and healthy, dislike physical activity [91], knowledge about the role that aberrant primary reflexes and an immature vestibular system plays, must also increase.

## Supporting information

S1 File Catching-up. Niklasson et al.
(SAV)

## References

[1] Orton ST. Reading, writing and speech problems in children. New York, NY: W.W. Norton & Co, Inc, 1937.

[2] Strauss AA, Lehtinen LE. Psychopathology and education of the brain-injured child. New York, NY: Grune & Stratton, Inc, 1947.

[3] Annell AL. School problems in children of average or superior intelligence: A preliminrary report. Journal of Mental Science 1949; 95:901–909. 15396383 10.1192/bjp.95.401.901

[4] Walton JN, Ellis E, Court SDM. Clumsy children: developmental apraxia and agnosia. Brain 1962; 85:603–612. 13998739 10.1093/brain/85.3.603

[5] Gordon N, McKinlay I. Helping clumsy children. London: Churchill Livingstone, 1980.

[6] Bejerot S. The relationship between poor motor skills and neurodevelopmental disorders. Dev Med Child Neurol 2011;53(9):779 doi: 10.1111/j.1469-8749.2011.04041.x 21752019 10.1111/j.1469-8749.2011.04041.x

[7] Rosenbaum DA. The Cinderella of psychology. The neglect of motor control in the science of mental life and behavior. American Psychologist 2005;60(4);308–317. doi: 10.1037/0003-066X.60.4.308 15943523 10.1037/0003-066X.60.4.308

[8] Gillberg C, Kadesjö B. ADHD with developmental coordination disorder In: Brown TE (ed). ADHD comorbidities. Handbook for ADHD complications in children and adults. Arlington, VA: American Psychiatric Publication, Inc, 2009:305–314.

[9] Gillberg C. The ESSENCE in child psychiatry: Early symptomatic syndromes elicting neurodevelopmental clinical examinations. Res Dev Disabil 2010;31(6):1543–1551. doi: 10.1016/j.ridd.2010.06.002 20634041 10.1016/j.ridd.2010.06.002

[10] Crespo-Eguilaz N, Magallón S, Narbona J. Procedural skills and neurobehavioral freedom. Front Hum Neurosci 2014 6 20;8:449 doi: 10.3389/fnhum.2014.00449 24999324 10.3389/fnhum.2014.00449 PMC4064662

[11] Kirby A, Sugden DA. Children with developmental coordination disorders. J R Soc Med 2007;100:182–186. doi: 10.1258/jrsm.100.4.182 17404341 10.1258/jrsm.100.4.182 PMC1847727

[12] Lingam R, Jongmans MJ, Ellis M, Hunt LP et al Mental health difficulties in children with developmental coordination disorder. Pediatrics 2012;129(4):e882–e891. doi: 10.1542/peds.2011-1556 22451706 10.1542/peds.2011-1556

[13] Bejerot S, Humble MB. Childhood clumsiness and peer victimization: a case-control study of psychiatric patients. BMC Psychiatry 2013;13:68 doi: 10.1186/1471-244X-13-68 23442984 10.1186/1471-244X-13-68 PMC3602183

[14] Rasmussen P, Gillberg C. Natural outcome of ADHD with developmental coordination disorder at age 22 years: A controlled, longitudinal, community-based study. J Am Acad Child Adolesc Psychiatry 2000;39(11): 1424–31. doi: 10.1097/00004583-200011000-00017 11068898 10.1097/00004583-200011000-00017

[15] Niklasson M, Rasmussen P, Niklasson I, Norlander T. Adults with sensorimotor disorders: Enhanced physiological and psychological development following specific sensorimotor training. Front Psychol 2015; 6:480 doi: 10.3389/fpsyg.2015.00480 25954233 10.3389/fpsyg.2015.00480 PMC4406001

[16] American Psychiatric Association. American Psychiatric Association: Diagnostic and statistical manual of mental disorders, 4^th^ edition Washington, DC: American Psychiatric Association 1994:53–55.

[17] American Psychiatric Association. Diagnostic and statistical manual of mental disorders, 5^th^ edition Arlington, VA: American Psychiatric Association 2013:74–77.

[18] Ahonen T, Kooistra L, Viholainen H, Cantell M. Developmental motor learning disability. A neuropsychological approach In: Dewey D, Tupper E (eds). Developmental motor disorders. A neuropsychological perspective. New York, NY:The Guildford Press, 2004:265–290.

[19] Blank R, Smits-Engelsman B, Polatajko H, Wilson P. European Academy of Childhood Disability (EACD): recommendations on the definition, diagnosis and intervention of developmental coordination disorder (long version). Dev Med Child Neurol 2012;54(1):54–93. doi: 10.1111/j.1469-8749.2011.04171.x 22171930 10.1111/j.1469-8749.2011.04171.x

[20] Cairney J. Developmental coordination disorder and its consequences: an introduction to the problem In: Cairney J (ed). Developmental coordination disorder and its consequences. Toronto: University of Toronto Press, 2015:5–30, 72.

[21] Ayd FJ Jr. Lexicon of psychiatry, neurology, and neurosciences. Philadelphia, PA: Lippincott Williams & Wilkins 2000:686.

[22] Wiener-Vacher SR, Hamilton DA, Wiener SI. Vestibular activity and cognitive development in children: perspectives. Front. Integr. Neurosci. 2013;7:92 doi: 10.3389/fnint.2013.00092 24376403 10.3389/fnint.2013.00092 PMC3858645

[23] Smits-Engelsman BCM, Blank R, van der Kaay AC, Mosterd-van der Meijs R et al Efficacy of interventions to improve motor performance in children with developmental coordination disorder: a combined systematic review and meta-analysis. Dev Med Child Neurol 2013;55(3):229–237. doi: 10.1111/dmcn.12008 23106530 10.1111/dmcn.12008

[24] Niklasson M, Niklasson I, Norlander T. Sensorimotor therapy: using stereotypic movements and vestibular stimulation to increase sensorimotor proficiency of children with attentional and motor difficulties. Percept. Mot. Skills 2009;108: 643–669. doi: 10.2466/PMS.108.3.643-669 19725302 10.2466/PMS.108.3.643-669

[25] Niklasson M, Niklasson I, Norlander T. Sensorimotor therapy: physical and psychological regressions contribute to an improved kinesthetic and vestibular capacity in children and adolescents with motor difficulties and concentration problems. Soc Behav Pers 2010; 38(3):327–346.

[26] Rasmussen P, Gillberg C, Waldenström E, Svenson B. Perceptual, motor and attentional deficits in seven-year-old children: neurological and neurodevelopmental aspects. Dev Med Child Neurol 1983; 25(3):315–333. 6873493 10.1111/j.1469-8749.1983.tb13765.x

[27] Besnard S, Lopez C, Brandt T, Denise P et al Editorial: The vestibular system in cognitive and memory processes in mammalians. Front Integr Neurosci 2015;9:55 doi: 10.3389/fnint.2015.00055 26617498 10.3389/fnint.2015.00055 PMC4639622

[28] Hämäläinen H, Takio F. Integrating auditory and visual asymmetry In: Hugdahl K, Westerhausen R. (eds.). The two halves of the brain. Information processing in the cerebral hemispheres. Cambridge, MA: The MIT Press 2010: 417–437.

[29] Sininger YS, Cone-Wesson B. Asymmetric cochlear processing mimics hemispheric specialization. Science 2004;305:1581 doi: 10.1126/science.1100646 15361617 10.1126/science.1100646

[30] Klatte M, Bergström K, Lachman T. Does noice affect learning? A short review on noise effects on cognitive performance in children. Front Psychol 2013;4:578 doi: 10.3389/fpsyg.2013.00578 24009598 10.3389/fpsyg.2013.00578 PMC3757288

[31] Tomlin D, Dillon H, Sharma M, Rance G. The impact of auditory processing and cognitive abilities in children. Ear Hear 2015;36(5):527–542. doi: 10.1097/AUD.0000000000000172 25951047 10.1097/AUD.0000000000000172

[32] Spellacy F, Blumstein S. Ear preference for language and non-language sounds: a unilateral brain function. J Aud Res 1970;10(4):349–354.

[33] Okamoto H, Stracke H, Ross B, Kakigi R et al Left hemispheric dominance during auditory processing in noisy environment. BMC Biol 2007;15(5);52 doi: 10.1186/1741-7007-5-52 18005401 10.1186/1741-7007-5-52 PMC2194668

[34] Jones PR, Moore DR, Amitay S. Development of auditory selective attention: Why children struggle to hear in noisy environments. Dev Psychol 2015;51(3):353–369. doi: 10.1037/a0038570 25706591 10.1037/a0038570 PMC4337492

[35] Norlander T, Moås L, Archer T. Noise and stress in primary and secondary school children: noise reduction and increased concentration ability through a short but regularly exercise and relaxation program. Sch Eff Sch Improv 2005;16:91–99.

[36] Faught BE, Hay JA, Cairney J, Flouris A. Increased risk for coronary vascular disease in children with developmental coordination disorder. J Adolesc Health 2005; 37(5):376–380. doi: 10.1016/j.jadohealth.2004.09.021 16227122 10.1016/j.jadohealth.2004.09.021

[37] Caçola P. Physical and mental health of children with developmental coordination disorder. Front. Public Health 2016;4:224 doi: 10.3389/fpubh.2016.00224 27822464 10.3389/fpubh.2016.00224 PMC5075567

[38] Rine RM. Growing evidence for balance and vestibular problems in children. Audiol Med 2009;7(3):138–142. doi: 10.1080/16513860903181447

[39] Largo RH, Caflisch JA, Hug F, Muggli K et al Neuromotor development from 5 to 18 years. Part 1: timed performance. Dev Med Child Neurol 2001;43:436–443. 11463173 10.1017/s0012162201000810

[40] Largo RH, Caflisch JA, Hug F, Muggli K et al Neuromotor development from 5 to 18 years. Part 2: associated movements. Dev Med Child Neurol 2001;43:444–453. 11463174 10.1017/s0012162201000822

[41] Gasser T, Rousson V, Caflisch J, Jenni OG. Development of motor speed and associated movements from 5 to 18 years. Dev Med Child Neurol 2010;52(3):256–63. doi: 10.1111/j.1469-8749.2009.03391.x 19583738 10.1111/j.1469-8749.2009.03391.x

[42] Benjumea JMC, Afonso JR, Hurtado JMR, Truan JCF. Assessment of motor coordination in students aged 6 to 11 years. J Phys Educ 2015; 15(4):765–774.

[43] Waber DP, De Moor C, Forbes PW, Almli CR et al The NIH MRI study of normal brain development: Performance of a population based sample of healthy children aged 6 to 18 years on a neuropsychological battery. J Int Neuropsychol Soc 2007; 13(5):729–746. doi: 10.1017/S1355617707070841 17511896 10.1017/S1355617707070841

[44] Konicarova J, Bob P. Retained primitive reflexes and ADHD in children. Act Nerv Super (Praha) 2012;54(3–4):135–138.

[45] Konicarova J, Bob P. Asymmetric tonic neck reflex and symptoms of attention deficit and hyperactivity disorder in children. Int J Neurosci 2013; 123(11):766–769. doi: 10.3109/00207454.2013.801471 23659315 10.3109/00207454.2013.801471

[46] Konicarova J, Bob P, Raboch J. Persisting primitive reflexes in medication-naÏve girls with attention-deficit and hyperactivity disorder. Neuropsychiatr Dis Treat 2013; 9:1457–1461. doi: 10.2147/NDT.S49343 24092983 10.2147/NDT.S49343 PMC3788695

[47] McPhillips M, Hepper PG, Mulhem G. Effects of replicating primary-reflex movements on specific reading difficulties in children: a randomised, double blind, controlled trial. The Lancet 2000;355(9203): 537–541. 10683004 10.1016/s0140-6736(99)02179-0

[48] Niklasson M, Niklasson I. Retraining for balance-Physiological test. Mönsterås, Sweden: Vestibularis, 2007a.

[49] Niklasson M, Niklasson I. Retraining for balance-Orientation and Balance test. Mönsterås, Sweden: Vestibularis, 2007b.

[50] Johansen KV. Lyd, horelse og sprogudvikling [Sound, hearing and the development of language]. Horsens, Denmark: Forlaget Aalokke a/s, 1993 [in Danish].

[51] Tallal P, Miller S, Holly Fitch R. Neurological basis of speech: a case for the preeminence of temporal processing In: Tallal P, Galaburda R, Llinas RR, von Euler C. Temporal information processing in the nervous system. Special reference to dyslexia and dysphasia. New York, NY: Annals of the New York Academy of Sciencies (No. 682),1993:27–47. 10.1111/j.1749-6632.1993.tb22957.x 7686725

[52] Conners CK. A teacher rating scale for use in drug studies with children. Am J Psychiatry, 1969;126:884–888. doi: 10.1176/ajp.126.6.884 4900822 10.1176/ajp.126.6.884

[53] Goyette CH, Conners CK, Ulrich RF. Normative data on revised Conners parent and teacher rating scales. J Abnorm Child Psychol 1978;6:221–236. 670589 10.1007/BF00919127

[54] Conners CK. Conners Rating Scales Manual, Conners Teacher Rating Scales, Conners Parent Rating Scales: Instruments for use with Children and Adolescents. North Tonawanda, NY: Multihealth Systems, 1990.

[55] Janols LO, von Knorring AL. Är medikamentell behandling motiverad vid hyperaktivitet hos barn? [Is drug therapy of hyperactive children justified?] Läkartidningen 1991;88:3057–3058 [in Swedish]. 1681156

[56] Gillberg C. Nordisk enighet om MBD-bedömning. Termen otidsenlig och olämplig. [Scandinavian unity on MBD assessment. The term is old-fashioned and unsuitable] Läkartidningen, 1991;88:713–717 [in Swedish]. 2002735

[57] Conners CK. Symptom patterns in hyperkinetic, neurotic, and normal children. Child Development 1970;41:667–682. doi: 10.2307/1127215

[58] Conners CK. Rating scales for use in drug studies with children. Psychopharmachology Bulletin 1973;9:24–29.

[59] Bergström M, Niklasson M, Niklasson I. Reason for training. Mönsterås, Sweden: Vestibularis, 1999.

[60] Cianfrani CA, Tsiakals JJ, West JE. ISO 9001:2008 explained. Milwaukee, WI: ASQ Quality Press, 2009.

[61] van Geert P. Nonlinear complex dynamical systems in developmental psychology In: Guastello SJ, Koopmans M, Pincus D (eds). Chaos and complexity in psychology. The theory of nonlinear dynamical systems. Cambridge, NY: Cambridge University Press, 2011:242.

[62] Wålinder R, Gunnarsson K, Runeson R, Smedje G. Physiological and psychological stress reactions in relation to classroom noise. Scand J Work Environ Health 2007;33(4):260–266. 17717617 10.5271/sjweh.1141

[63] Lacerda AB, Goncalves CG, Lacerda G, Lobato DC et al Childhood hearing health: educating for prevention of hearing loss. Int Arch Otorhinolaryngol 2015;19(1):16–21. doi: 10.1055/s-0034-1387810 25992146 10.1055/s-0034-1387810 PMC4392508

[64] Peterson ML, Christou E, Rosengren KS. Children achieve adult-like sensory integration during stance at 12-years-old. Gait & Posture 2006;23:455–463. doi: 10.1016/j.gaitpost.2005.05.003 16002294 10.1016/j.gaitpost.2005.05.003

[65] Smith AW, Ulmer FF, Wong del P. Gender differences in postural stability among children. J Hum Kinet 2012;33:25–32. doi: 10.2478/v10078-012-0041-5 23487417 10.2478/v10078-012-0041-5 PMC3588681

[66] Steindl R, Kunz K, Schrott-Fischer A, Scholtz AW. Effect of age and sex on maturation of sensory systems and balance control. Dev Med Child Neurol 2006;48(6):477–482. doi: 10.1017/S0012162206001022 16700940 10.1017/S0012162206001022

[67] Odenrick P, Sandstedt P. Development of postural sway in the normal child. Hum Neurobiol 1984;3(4):241–244. 6526710

[68] Hirabayashi S, Iwasaki Y. Developmental perspective of sensory organization on postural control. Brain Dev 1995;17(2):111–113. 7542846 10.1016/0387-7604(95)00009-z

[69] Teicher JD. Preliminary survey of motility in children. J Nerv Ment Dis 1941;94(3): 277–304. doi: 10.1097/00005053-194109000-00002

[70] Peters JE, Romine JS, Dykman RA. A special neurological examination of children with learning disabilities. Dev Med Child Neurol 1975;17(1): 63–78. 1123123 10.1111/j.1469-8749.1975.tb04959.x

[71] Brodal P. The central nervous system. Structure and function. Oxford: Oxford University Press, 2004: 275.

[72] Kimura D. From ear to brain. Brain and Cognition 2011;76:214–217. doi: 10.1016/j.bandc.2010.11.009 21236541 10.1016/j.bandc.2010.11.009

[73] Adams JA. Historical review and appraisal of research on the learning, retention, and transfer of human motor skills. Psychol Bull 1987(1);101: 41–74.

[74] Cohen J. Statisticasl power analysis for the behavioral sciences. Hillsdale, NJ: Lawrence Erlbaum Associates 1988.

[75] Holt KS. Child development. London: Butterworth-Heinemann, 1991: 4–5.

[76] Missiuna C., Moll S., King G., Stewart D., Macdonald K. Life experiences of young adults who have coordination difficulties. Can J Occup Ther 2008;75(3):157–166. doi: 10.1177/000841740807500307 18615927 10.1177/000841740807500307

[77] Snapp-Childs W., Mon-Williams M., Bingham G.P. A sensorimotor approach to the training of manual actions in children with DCD. J Child Neurol 2013;28(2):2014–212. doi: 10.1177/0883073812461945 23076426 10.1177/0883073812461945 PMC3695700

[78] Adams RM, Kocsis JJ, Estes RE. Soft neurological signs in learning-disabled children and controls. Am J Dis Child 1974;128(5):614–618. 4417177 10.1001/archpedi.1974.02110300024004

[79] Touwen BCL. The meaning and value of soft signs in neurology In: Tupper DE (ed). Soft neurological signs. Orlando, FL: Grune & Stratton, 1987:291.

[80] Gottlieb G, Wahlsten D, Lickliter R. The significance of biology for human development: A developmental psychobiological systems view In: Lerner RM (ed). Handbook of childpsychology, 6:th edition Hoboken, NJ: John Wiley & Sons, Inc, 2006:213.

[81] Mancini VO, Rigoli D, Cairney J, Roberts LD, Piek JP. The elaborated environmental stress hypothesis as a framework for understanding the association between motor skills and internalizing problems: a mini review. Front Psychol 2016; 7:239 doi: 10.3389/fpsyg.2016.00239 26941690 10.3389/fpsyg.2016.00239 PMC4763061

[82] Andreasen NC, Carpenter W, Kane JM, Lasser RA, Marder SR, Weinberger DR. Remission in schizophrenia: proposed criteria and rationale for consensus. Am J Psychiatry 2005;162(3):441–449. doi: 10.1176/appi.ajp.162.3.441 15741458 10.1176/appi.ajp.162.3.441

[83] Helldin L, Kane JM, Hjärthag F, Norlander T. The importance of cross-sectional remission in schizophrenia for long-term outcome: a clinical perspective study. Schizophr Res 2009;115(1):67–73. doi: 10.1016/j.schres.2009.07.004 19666215 10.1016/j.schres.2009.07.004

[84] Harvey PD, Helldin L, Bowie CR, Heaton RK, Olsson AK, Hjärthag F, Norlander T, Patterson TL. Performance-based measurement of functional disability in schizophrenia: a cross-national study in the United States and Sweden. Am J Psychiatry 2009;166(7):821–827. doi: 10.1176/appi.ajp.2009.09010106 19487393 10.1176/appi.ajp.2009.09010106 PMC3667206

[85] Norlander T, Ernestad E, Moradiani Z, Nordén T. Perceived feeling of security: A candidate for assessing remission in borderline patients? The Open Psychology Journal 2015;8:146–152.

[86] Weiden P, Aquila R, Standard J. Atypical antipsychotic drugs and long-term outcome in schizophrenia. J Clin Psych 1996;57(Suppl 11):53–60. 8941171

[87] BrÜne M, Belsky J, Fabrega H, Feierman JR et al The crisis in psychiatry-insights and prospects from evolutionary theory. World Psychiatry 2012;11(1):55–57. 22295011 10.1016/j.wpsyc.2012.01.009 PMC3266750

[88] Panksepp J. Will better psychiatric treatments emerge from top-down or bottom-up neuroscientific studies of affect? World Psychiatry 2014;13(2):141–142. doi: 10.1002/wps.20120 24890060 10.1002/wps.20120 PMC4102280

[89] Pollak SD. Developmental psychopathology: recent advances and future challenges. World Psychiatry 2015;14(3):262–269. doi: 10.1002/wps.20237 26407771 10.1002/wps.20237 PMC4592638

[90] Hallal PC, Martins RC, Ramirez A. The Lancet physical activity observatory: promoting physical activity worldwide. Lancet 2014; 384 (9942): 471–472. doi: 10.1016/S0140-6736(14)61321-0 25110267 10.1016/S0140-6736(14)61321-0

[91] Bergman A., and Norlander T.”Hay sacks anonymous”: Living in the shadow of the unidentified. Psychological aspects of physical inactivity from a phenomenological perspective. The Qualitative Report 2005;10(4): 795–816.

